# Supplementary material for: Gibberellic Acid Inhibits Dendrobium nobile—Piriformospora Symbiosis by Regulating the Expression of Cell Wall Metabolism Genes
Source: Biomolecules. 2023 Nov 14;13(11):1649. doi: 10.3390/biom13111649 (PMC10669577; doi:10.3390/biom13111649)
Supplement: Supplementary file 1 [file biomolecules-13-01649-s001.zip › Supplementary information.pdf]

Supplementary information

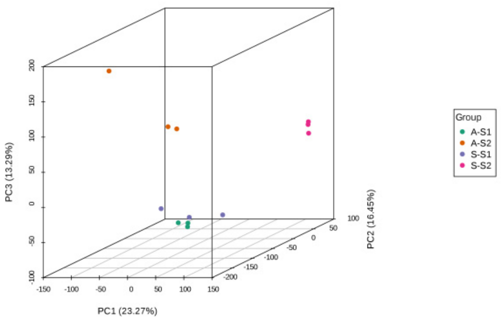

Figure S1. The principle component analysis (PCA) of all samples.

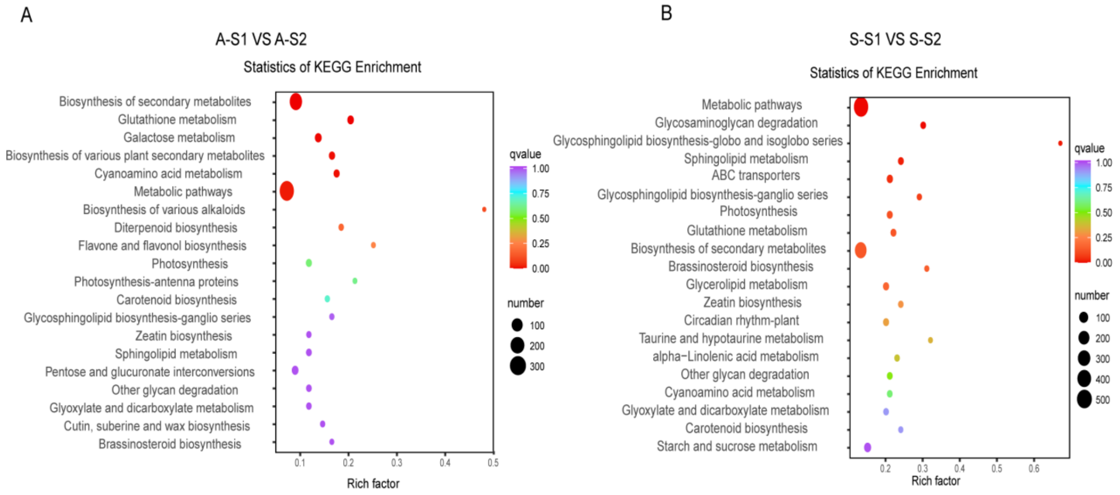

Figure S2. The analysis of KEGG enrichment in the comparison group A-S1 VS S-S1 and group A-S2 VS S-S2.

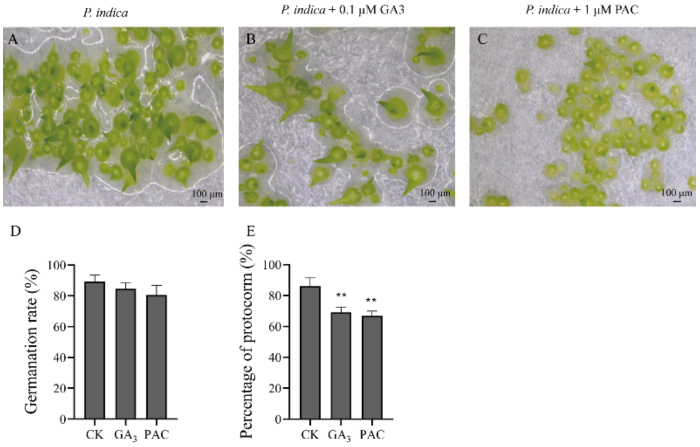

Figure S3 Effect of exogenous GA_3 and PAC on *D. nobile* seeds germinate progress

A-C: morphological characters of symbiotic germination inoculated with *P. indica* at different treatment with GA3 and PAC. D: effect on seed germination of GA_3 and PAC treatments. E: effect on protocorm formation of GA_3 and PAC treatments, five biological replicates were performed, t-test (two tailed).

Table S1. Overview of transcriptome sequencing and de novo assembly results.

Table S2. Mapping clean RNA-seq reads to the reference genome of *D. nobile.*

Table S3. Annotated genes related to GA metabolism and signaling.

Table S4. Annotated genes related to IAA metabolism and signaling.

Table S5. Annotated genes related to cell wall metabolism.

Table S6. Primers used for qPCR assay.
